# Exploring the clinical relevance of vital signs statistical calculations from a new-generation clinical information system

**DOI:** 10.1038/s41598-023-40769-3

**Published:** 2023-09-12

**Authors:** Juan Ignacio Muñoz-Bonet, Vicente Posadas-Blázquez, Laura González-Galindo, Julia Sánchez-Zahonero, José Luis Vázquez-Martínez, Andrés Castillo, Juan Brines

**Affiliations:** 1 Paediatric Intensive Care Unit, Hospital Clínico Universitario, Av. Blasco Ibáñez 17, 46010 Valencia, Spain; 2 Department of Paediatrics, Obstetrics, and Gynaecology, University of Valencia, Valencia, Spain; 3 Paediatric Intensive Care Unit, Hospital Universitario Ramón y Cajal, Madrid, Spain; 4 Paediatric Technological Innovation Department, Foundation for Biomedical Research of Hospital Niño Jesús, Madrid, Spain

**Keywords:** Medical research, Health care, Paediatrics, Information technology

## Abstract

New information on the intensive care applications of new generation ‘high-density data clinical information systems’ (HDDCIS) is increasingly being published in the academic literature. HDDCIS avoid data loss from bedside equipment and some provide vital signs statistical calculations to promote quick and easy evaluation of patient information. Our objective was to study whether manual records of continuously monitored vital signs in the Paediatric Intensive Care Unit could be replaced by these statistical calculations. Here we conducted a prospective observational clinical study in paediatric patients with severe diabetic ketoacidosis, using a Medlinecare^®^ HDDCIS, which collects information from bedside equipment (1 data point per parameter, every 3–5 s) and automatically provides hourly statistical calculations of the central trend and sample dispersion. These calculations were compared with manual hourly nursing records for patient heart and respiratory rates and oxygen saturation. The central tendency calculations showed identical or remarkably similar values and strong correlations with manual nursing records. The sample dispersion calculations differed from the manual references and showed weaker correlations. We concluded that vital signs calculations of central tendency can replace manual records, thereby reducing the bureaucratic burden of staff. The significant sample dispersion calculations variability revealed that automatic random measurements must be supervised by healthcare personnel, making them inefficient.

## Introduction

There is a broad consensus that future healthcare will require the intensive use of information technology to acquire, store, process, analyse, and use the information extracted from medical data^1,2^. The Anaesthesia and Critical Care specialities are the most technical and data-driven medical environments and so the development of new approaches for the integration and use of the data generated in these fields is almost mandatory^1–7^. However, the basic approach to data collection and management has remained largely unchanged over the past 40 years^2^. Indeed, it is now becoming especially difficult to integrate and take advantage of the information provided by patient bedside monitoring and treatment devices^2^. In critical patients, this information is traditionally registered by nursing staff, who collect the most representative values from each period, usually once an hour^8^. This chart is used as the baseline by which patient evolution is then assessed^9^.

Given that these patient monitoring charts are costly to prepare, some centres have replaced this type of record keeping with automatic random data collection by different clinical information systems (CIS)^2,8^. These CIS usually collect one data point per parameter every 15, 30, or 60 min^8,10^. This means that more than 99% of the information generated at the bedside is lost with no possibility reaping the benefits that its exploitation could mean for patients and the healthcare system^2,4,11,12^. In addition, CIS do not provide any processing or analysis of the information obtained^2^. Thus, although some of these systems provide access to up to 1 piece of data per minute, the volume of data makes its routine manual evaluation by healthcare personnel difficult^13^ and most of the information is still lost. Because of all the above, Intensive Care Unit (ICU) teams continue to express frustration with the current patient data representation by CIS^14^.

Therefore, a new approach to the use of information generated at the bedside is required to prevent this data loss and to facilitate its clinical use^4,8,11^. The main objective of this new approach is to apply big-data principles (volume, velocity, variety, veracity, and data value) to the concept of personalised medicine. In this sense, information has already been published about the application of high-density data CIS (HDDCIS) in intensive care contexts, both in development studies^2,3,13,15–17^ and as original research^4,8,12,18–23^. Although the use of HDDCIS is not yet common in ICUs, in the future they will help revolutionise the monitoring of vital signs as we currently know it. In this context, Matam et al. analysed the technical difficulties and feasibility of adapting HDDCIS to their Paediatric Intensive Care Unit (PICU) and created a machine learning system that predicts cardiac arrest in children^15,24^. In turn, Brossier et al. argued for the importance of these systems being able to indefinitely store all the monitoring data for ‘perpetual patients’ as well as their advantages for the development of clinical decision support systems (CDSS)^4,25,26^.

Furthermore, the development of artificial intelligence (AI) is likely to strongly impact intensive care medicine. Indeed, one of the main lines of ongoing research for many groups working in this field involves the use of AI to give HDDCIS the ability to detect clinically important events. For example, in the field of mechanical ventilation, systems that predict accidental extubation^27^ or that protect the lungs by avoiding volutrauma^28^ are already available. The detection of events in the operating room during anaesthesia has also been analysed by leveraging the information contained in databases that integrate information from different clinical records such as electrocardiograms, oxygen saturation, heart rate, and bispectral index results^29,30^. Thus, the integration of vital signs records has already allowed the creation of machine learning systems capable of detecting, in real time, events such as acute hypotension^31^, responses to vasoactive drugs^32^, or the need for massive transfusion in the operating room^33^. In most cases, the AI technology used to detect events is based on the registration of vital signs data and leverages pre-defined scores. Among these indices, it is worth highlighting the Inadequate Oxygen Delivery Index (IDO2-Index) that warns of the risk of adverse events such as cardiorespiratory arrest, the development of enterocolitis, need for extracorporeal membrane oxygenation (ECMO), or renal replacement therapy in children undergoing cardiac surgery^34^.

In this line, our team has several years of clinical experience with the use of this technology^35^ and we have been able to verify the clinical usefulness of the hourly statistical calculations of vital signs provided by the HDDCIS^6^. However, the use of these calculations has not yet been validated. Thus, the objective of this current work was to study whether manual records of continuously monitored vital signs can be replaced by the statistical calculations of central tendency provided by an HDDCIS.

## Materials and methods

This prospective observational clinical study was conducted in children with severe diabetic ketoacidosis consecutively admitted to the PICU at the University Clinical Hospital of Valencia (a 6-bed multivalent unit in a tertiary hospital), between 2017 and 2020. The inclusion criteria were (1) blood glucose exceeding 11.1 mmol/L; (2) ketonemia greater than 1 mmol/L; (3) bicarbonate less than 8 mEq/L; and (4) base excess exceeding − 20 mEq/L. We selected these patients for their special clinical characteristics. Upon admission, they presented a serious metabolic alteration that affected their vital signs but without associated respiratory, cardiocirculatory, or other pathologies or confounding factors such as the requirement for mechanical ventilation, administration of inotropic drugs, sedatives, or analgesics, among others^9,22^. Thus, with appropriate treatment, their vital signs returned to normal within hours, therefore making them good models to assess clinical evolutionary changes and to compare different study parameters.

This study was conducted in accordance with the amended Declaration of Helsinki and was approved by the Ethics Committee at the Biomedical Research Institute INCLIVA (grant number 2017/022). Informed consent was obtained from the families of the patients. We used the Medlinecare^®^ HDDCIS (Medical Online Technology S.L., Valencia, Spain) which receives and transmits information from bedside monitoring and treatment equipment at a rate of 1 data point per monitored parameter every 3–5 s, (720–1200 data points per hour), including equipment from multiple manufacturers. This allowed us to continuously monitor the clinical status of our patients in real time. In addition, the HDDCIS stores data and automatically calculates hourly statistical indicators of the sample central trends (mean, mode, and median) and dispersion as the maximum (99th percentile), and minimum (1st percentile) values. These calculations were performed in the first few minutes of each new hour.

The data collected during patient admissions to the PICU are shown in Table 1. The nursing staff did not have access to the information provided by the HDDCIS or knowledge of the objective of this current study. We used pulse oximetry technology from Masimo Corp. (Irvine, CA) and collected oxygen saturation (SpO_2_) and pulse oximetry heart rate (pHR) data using disposable fingertip probes. We also collected electrocardiography heart rate (eHR) and respiratory rate data measured by impedance (iRR) using Infinity Delta XL multiparameter monitors from Dräger Medical (Lübeck, Germany). To uncover whether the clinical evolution of the patients could affect the correlation study of the parameters, the data series were divided into two groups according to the level of bicarbonate present in the blood of the patients: the *severe acidosis group* versus the *improvement group*, with the bicarbonate cut-off point being ≥ 10 meq/L.

**Table 1:** Data collected during patient admissions to the Paediatric Intensive Care Unit.

1. PICU nursing hourly manual records of continuously monitored parameters			
Heart rate (nHR)		Respiratory rate (nRR)	Oxygen saturation (nSpO_2_)
2. Automatic hourly statistics calculations provided by the HDDCIS			
Heart rate Measured by pulse oximetry (pHR) Measured by electrocardiography (eHR)		Respiratory rate measured by impedance (iRR)	Oxygen saturation measured by pulse oximetry (SpO_2_)
3. Evolutionary biochemical analysis in venous blood			
Glycaemia (mmol/L)		Ketonemia (mmol/L)	Lactacidemia (mmol/L)
4. Evolutionary acid–base balance in venous blood			
pH	Partial pressure of carbon dioxide (PCO_2_) (mmHg)	Bicarbonate (mEq/L)	Base excess (mmol/L)

*PICU* Paediatric Intensive Care Unit, *n prefix* records made manually by nurses.

### Statistical analysis

SPSS software (v26.0 IBM Corp., Armonk, NY) was used to carry out the statistical analyses. The relationships between continuous variables were evaluated employing the Pearson or Spearman correlation coefficient, depending on the data distribution (after assessing the latter using the Kolmogorov–Smirnov test). We also used Student *t*-tests for related samples, Lin’s concordance correlation coefficient (CCC) to assess the data agreement^36^, and Bland–Altman plots for multiple measurements per patient, as calculated with MedCalc software^37^. Analysis of variance (ANOVA) was used to study the relationship between the continuous variables of each group. However, when statistically significant results failed to meet the ANOVA assumptions, we resorted to an alternative robust test (Welch’s test). The significance level threshold was set at an alpha of 0.05 in all cases.

## Results

Thirty-two consecutive patients (21 boys and 11 girls) aged 9.1 ± 4.3 years were included in this study cohort. Around 2,761,000 measurements were used to obtain 1027 hourly statistical calculations for pHR, eHR, and SpO_2_, as well as 745 calculations of iRR. Simultaneous hourly nursing records (denoted with the ‘n’ prefix) were also collected for the heart rate (nHR = 1025), respiratory rate (nRR = 716), and oxygen saturation (nSpO_2_ = 980). In addition, 212 periodic determinations of glycaemia, ketonemia, lactate, and acid–base balance were also collected. Upon admission, the patients presented the following blood analytical test data: blood glucose = 25.8 ± 7.6 mmol/L, ketonemia = 5.3 ± 1.7 mmol/L, lactate = 2.2 ± 0.9 mmol/L, pH = 7.05 ± 0.1, PCO_2_ = 19.6 ± 7.5 mmHg, bicarbonate = 5.6 ± 2.9 mEq/L, and base excess =  − 24.7 ± 4.7 mEq/L.

**Figure 1:**
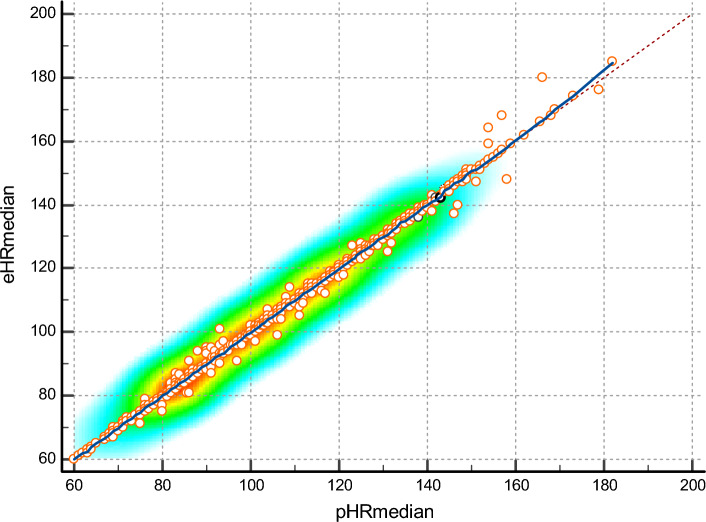
Scatter diagram of the automatic hourly calculation of the ‘median heart rate’ obtained by pulse oximetry (pHR-median) and electrocardiography (eHR-median). Note how the line of equality and the trend line (blue) are identical (locally weighted scatterplot smoothing span = 0%; concordance correlation coefficient [CCC] = 0.9983; Pearson *p* for precision = 0.9984; bias correction factor for accuracy = 1 [95% CI 0.9982–0.9986]; *p* < 0.0001). Similar results were obtained for the central tendency calculations for the ‘mean heart rate’ and ‘modal heart rate’ (CCC > 0.99).

The hourly calculations of the central tendency for pHR and eHR were identical and their CCC was almost perfect (Fig. 1). These calculations showed identical values, very strong correlations, and a substantial CCC with the nHR data (Table 2). Indeed, both the nHR and the central tendency calculations for pHR and eHR showed the same moderately significant correlations with the evolution of the acid–base balance. For example, for pH, *r* = − 0.51 for nHR and *r* = − 0.5 for pHR, for PCO_2_, *r* = − 0.53 and − 0.55, respectively, for bicarbonate *r* = − 0.55 and − 0.55, respectively, and for base excess, *r* = − 0.56 and − 0.56, respectively.

**Table 2:** Descriptive statistics, correlations, and concordances between the manual nursing records and automatic statistical calculations.

	Mean	*SD*	Correlation	Concordance correlation coefficient			Paired student *t*-test
				CCC	Precision	Accuracy	
nHR (*N* = 1025)	107	22.8	(Spearman)		(Pearson)	Bias correction factor	
pHR-mode	106	23.3	0.982	0.981	0.981	0.999	*p* < 0.001
pHR-mean	107	23	0.980	0.981	0.981	1	*p* < 0.001
pHR-median	107	23.1	0.982	0.982	0.982	1	*p* = 0.964
pHR-maximum	122	24.9	0.928	0.765	0.921	0.84	*p* < 0.001
pHR-minimum	97	22.9	0.935	0.839	0.915	0.918	*p* < 0.001
eHR-mode	106	23.4	0.978	0.977	0.979	0.999	*p* < 0.001
eHR-mean	107	23.2	0.977	0.977	0.977	1	*p* = 0.01
eHR-median	106	23.2	0.980	0.98	0.98	1	*P* = 0.112
eHR-maximum	121	24.5	0.923	0.768	0.916	0.838	*p* < 0.001
eHR-minimum	97	23.7	0.967	0.891	0.961	0.927	*p* < 0.001
nRR (*n* = 716)	23	7.3	(Spearman)		(Pearson)		
iRR-mode	23	9.5	0.739	0.54	0.56	0.964	*p* = 0.055
iRR-mean	26	8.7	0.636	0.501	0.542	0.925	*p* < 0.001
iRR-median	24	8.9	0.706	0.543	0.558	0.972	*p* < 0.001
iRR-maximum	44	18.7	0.324	0.086	0.256	0.337	*p* < 0.001
iRR-minimum	17	5.6	0.644	0.404	0.632	0.639	*p* < 0.001
nSpO_2_ (*n* = 980)	98.8	1.3	(Spearman)		(Pearson)		
SpO_2_-mode	98.8	1.4	0.79	0.817	0.819	0.998	*p* = 0.004
SpO_2_-mean	98.6	1.3	0.759	0.784	0.789	0.994	*p* < 0.001
SpO_2_-median	98.8	1.4	0.775	0.816	0.817	0.999	*p* = 0.172
SpO_2_-maximum	99.7	0.7	0.6	0.418	0.659	0.633	*p* < 0.001
SpO_2_-minimum	96.2	3.9	0.523	0.081	0.181	0.447	*p* < 0.001

*nHR* hourly heart rate recorded by nurses, *pHR* automatic hourly calculation of the heart rate through pulse oximetry, *eHR* automatic hourly calculation of the heart rate through electrocardiography, *nRR* hourly respiratory rate recorded by nurses, *iRR* automatic hourly calculation of the respiratory rate through impedance, *nSpO*_*2*_ hourly oxygen saturation recorded by nurses, *SpO*_*2*_ automatic hourly oxygen saturation calculation through pulse oximetry. The Student *t*-test results indicated that the best correlation with the nursing values was obtained with the median for heart rate and oxygen saturation and with the mode for the respiratory rate.

**Figure 2:**
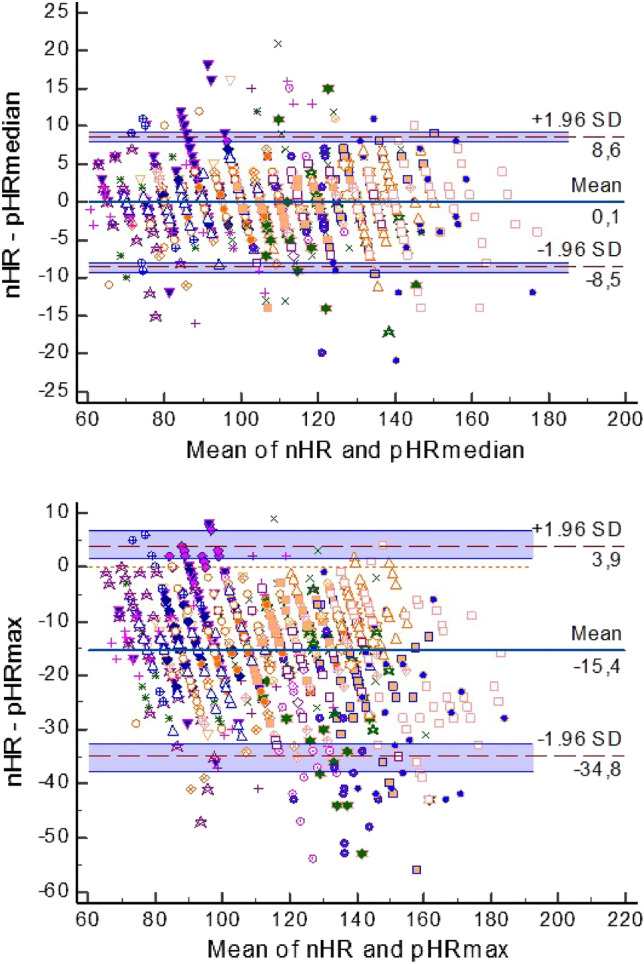
Bland–Altman plot for multiple measurements per patient of the heart rate measured hourly by nursing staff (nHR) alongside the hourly automatic calculation of the median heart rate (pHR-median) and maximum heart rate (pHR-max). The sample size was *N* = 1025.

The central tendency calculations for the iRR and SpO_2_ behaved in a similar way, with identical or remarkably similar values and strong correlations with the nursing staff references (except for the mean iRR, which showed a moderate correlation). The maximum and minimum hourly calculations differed from their nursing references and from the central tendency calculations in all the variables by showing weaker correlations. These differences were clinically significant for heart rate (HR) and respiratory rate (RR). The concordance study results are shown in Tables 2 and 3 and in Fig. 2.

**Table 3:** The difference between the manual data recordings by nurses and by the automatic statistical calculations.

Difference nHR − pHR (*n* = 1025)	Mode	Mean	Median	Maximum	Minimum
Mean	0.86	− 0.68	0.05	− 15.4	9.9
P (H_0_: mean = 0)	< 0.001	< 0.001	0.964	< 0.001	< 0.001
Lower limit (95% CI) of agreement	− 8	− 9.5	− 8.5	− 34.8	− 8.7
Upper limit (95% CI) of agreement	9.7	8.2	8.6	3.9	28.5

Difference nRR − iRR (*n* = 716)	Mode	Mean	Median	Maximum	Minimum
Mean	0.63	− 2.9	− 1.1	− 20.1	6.3
P (H_0_: mean = 0)	0.055	< 0.001	0.003	< 0.001	< 0.001
Lower limit (95% CI)	− 15.4	− 18.5	− 16.6	− 56.5	− 5.6
Upper limit (95% CI)	16.7	12.7	14.5	16.3	18.2

Difference nSpO_2_ − SpO_2_ (*n* = 983)	Mode	Mean	Median	Maximum	Minimum
Mean	− 0.12	0.09	− 0.07	− 0.88	2.52
P (H_0_: mean = 0)	0.004	< 0.001	0.172	< 0.001	< 0.001
Lower limit (95% CI)	− 1.72	− 1.58	− 1.67	− 2.9	− 5.06
Upper limit (95% CI)	1.49	1.75	1.52	1.14	10.1

*nHR* heart rate recorded by nurses, *nRR* respiratory rate recorded by nurses, *nSpO*_*2*_ oxygen saturation recorded by nurses, *pHR* hourly calculation of the pulse-based heart rate, *iRR* hourly calculation of the respiratory rate by impedance, *SpO*_*2*_ hourly calculation of the oxygen saturation. Data calculated using the Bland–Altman method. The differences between the nHR and electrical heart rate (eHR) are not provided because of their similarity to those of the pHR.

In the comparison by groups (severe acidosis versus improvement), blood glucose, ketonemia, lactate, and acid–base balance measurements all improved, reaching levels of clinically and statistically significant differences (*p* < 0.001). HR and RR were also significantly improved for all variables from both the clinical and statistical perspective, except for the maximum iRR (Tables 4 and 5). In contrast, SpO_2_ decreased with improving acidosis, although these differences did not reach the level of clinical significance (Table 4).

**Table 4:** Descriptive statistics and group comparisons for the heart rate and oxygen saturation.

Heart rate parameters	Mean	*SD*	SpO_2_ parameters	Mean	*SD*
Severe acidosis group (bicarbonate < 10 meq/L)					
(*n* = 226)			(*n* = 216)		
nHR	120*	20.6	nSpO_2_	99.3*	1.1
pHR-mode	119*	21.5	SpO_2_ mode	99.6*	0.8
pHR-mean	120*	20.8	SpO_2_ mean	99.4*	0.8
pHR-median	120*	21.1	SpO_2_ median	99.5*	0.8
pHR-maximum	135*	22.9	SpO_2_ maximum	99.9*	0.3
pHR-minimum	111*	21.2	SpO_2_ minimum	97.1^#^	4.0
Clinical improvement group (bicarbonate ≥ 10 meq/L)					
(*n* = 799)			(*n* = 764)		
nHR	103*	21.9	nSpO_2_	98.6*	1.4
pHR-mode	102*	22.5	SpO_2_ mode	98.6*	1.5
pHR-mean	104*	22.3	SpO_2_ mean	98.5*	1.3
pHR-median	103*	22.4	SpO_2_ median	98.6*	1.4
pHR-maximum	118*	24.6	SpO_2_ maximum	99.6*	0.8
pHR-minimum	93*	21.8	SpO_2_ minimum	96.0^#^	3.9

*nHR* heart rate recorded by nurses, *pHR* hourly calculation of the pulse-based heart rate, *nSpO*_*2*_ oxygen saturation recorded by nurses. There were statistically significant differences between the groups for all the monitored parameters (*) *p* < 0.001, (#) *p* = 0.001. Note how there was a significant intragroup oscillation in the HR (the maximum and minimum values differed by ≈ 25 bpm). The evolution of the electrical heart rate (eHR) by groups was not provided because it was identical to that of the pHR.

**Table 5:** Descriptive statistics, concordances, and comparisons of the respiratory rate by groups.

Parameters	Mean	*SD*	Concordance correlation coefficient			Asymmetry	Kurtosis
			CCC	Precision	Accuracy		
Severe acidosis group (bicarbonate < 10 meq/L, *n* = 182)							
nRR	27*	8.6		(Pearson)	Bias correction factor	0.64^a^	0.39^c^
iRR-mode	25*	8.4	0.856	0.875	0.978	0.77^a^	0.07^c^
iRR-mean	28^#^	8.4	0.829	0.836	0.991	0.47^a^	− 0.4^c^
iRR-median	26^#^	8.3	0.878	0.882	0.996	0.67^a^	− 0.08^c^
iRR-maximum	44	17.6	0.196	0.443	0.442	0.96^a^	0.85^c^
iRR-minimum	20*	6.5	0.534	0.784	0.681	0.88^a^	0.46^c^
Improvement group (bicarbonate ≥ 10 meq/L, *n* = 534)							
nRR	22*	6.3		(Pearson)	Bias correction factor	1.05^b^	2.3^d^
iRR-mode	22*	9.5	0.393	0.429	0.917	3.7^b^	19.2^d^
iRR-mean	26^#^	8.7	0.353	0.409	0.863	2.15^b^	7.44^d^
iRR-median	24^#^	8.9	0.388	0.422	0.921	2.82^b^	12^d^
iRR-maximum	43	18.8	0.053	0.185	0.285	1.03^b^	0.26^d^
iRR-minimum	16*	4.7	0.272	0.473	0.576	1.25^b^	5.1^d^

*nRR* hourly nursing record of respiratory rate, *iRR* automatic hourly calculation of respiratory rate measured by impedance. Note (1) the marked intragroup oscillation of the RR (≈ 25 bpm); (2) the asymmetry and kurtosis values were close to the normal distribution in the group of patients with severe acidosis. In contrast, in the improved group, the nRR and, especially, the central tendency calculations presented high asymmetries and kurtosis, showing a leptokurtic curve with a distribution tail stretching to the right for values above the mean (the true measurements of RR were grouped into the leptokurtic values, while the movement artifacts, which were more frequent in the improvement group, were grouped into the tail on the right); (3) the poorest correlation and concordance values were obtained for the iRR-maximum (which included motion artifacts); (4) as expected, the RR decreased as acidosis improved, with the differences between the groups being clinically and statistically significant for all the parameters, except for the iRR-maximum. (*) *p* < 0.001, (#) *p* = 0.001. Deviation error: (a) 0.18, (b) 0.1, (c) 0.36, (d) 0.21.

## Discussion

### The physiological basis and main characteristics of high-density data clinical information systems

**Figure 3:**
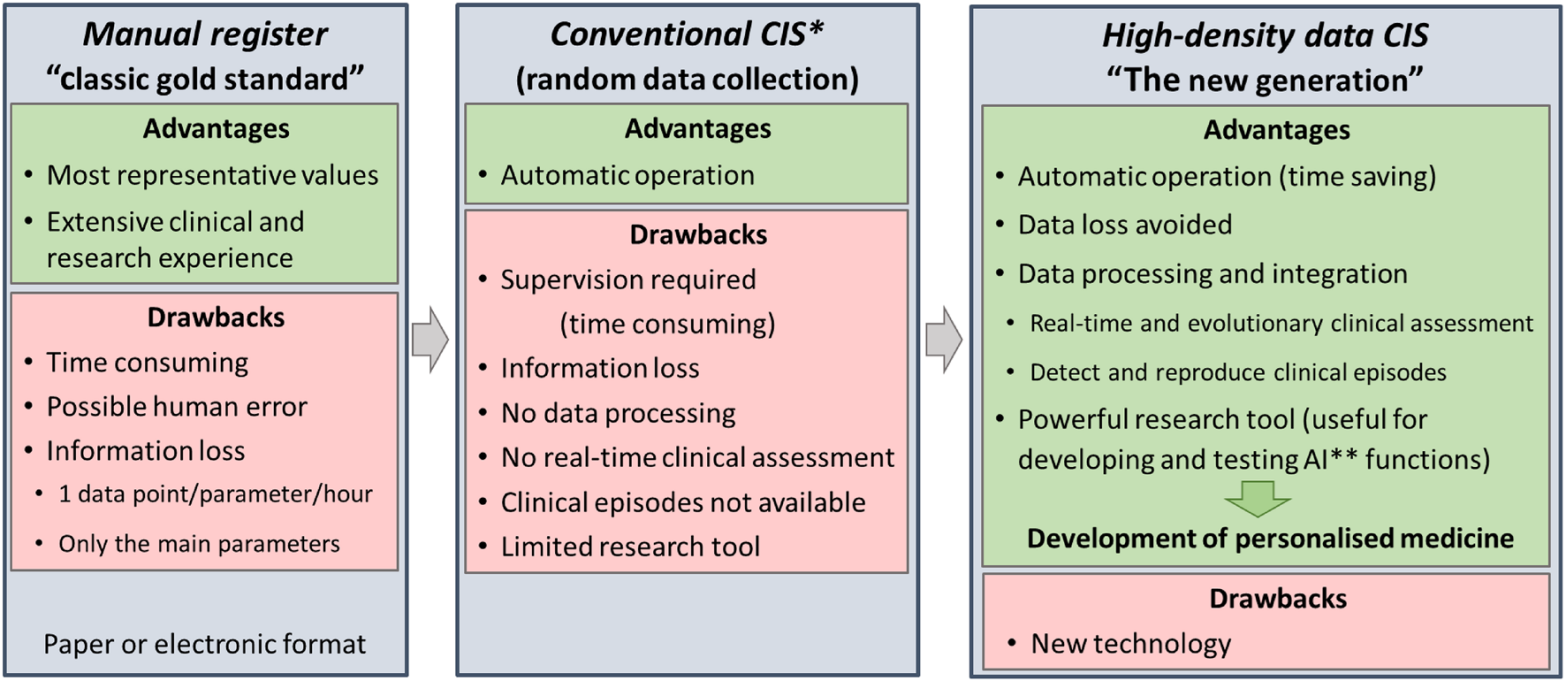
Record of information provided by patient bedside monitoring and treatment devices. (*) *CIS* clinical information systems, (**) *AI* artificial intelligence.

Drs. Horvat and Ogoe pointed out that “with the conventional approach, the patient data generated in the ICU are continually reduced to summary information, which has the risk of over-distilling the relevant and complex physiology of these patients”. Thus, these authors and others believe that the use of high-frequency data systems, “may further our understanding of intensive care physiology and eventually support the development of more individualised therapeutic regimens”^5,9^. As described in the introduction, this technology is already being applied in hospitals all over the world. More specifically, four basic properties characterise HDDCIS (Fig. 3) as follows:

1. Sampling frequency. Although this can differ according to the parameters recorded, for vital signs and other fundamental parameters, this frequency must be less than 1 data point every 10 s. However, many current systems capture these values at intervals of minutes, which is insufficient to follow patient evolution in real time or to detect and evaluate clinical events.
2. Multidevice capacity. Anaesthesia and critical care environments are complex by nature and are home to countless medical devices for patient monitoring and treatment. Thus, to include any parameter of clinical interest, it must be possible to capture data, at a high sampling frequency, from different pieces of medical equipment. To do this, the problem of device synchronisation must be solved.
3. Information processing. This is important both in terms of real-time care functionality, as well as in the evaluation of clinical evolution based on historical data^6^. As Sun et al. stated, “we must develop a data acquisition system that facilitates the access and review of historical data for medical personnel. Furthermore, acquired data should be […] presented to clinical staff in such a manner that supports clinical decision making”^13^. Thus, improving this information processing will directly enhance care provider wellbeing, patient outcomes, and quality of care^14^. Moreover, HDDCIS are of great educational utility and can also help improve quality of care because they can fully detect clinical episodes as they happen, at high resolution, and can also reproduce them for later analysis (see Supplementary Figs. S1–S3).
4. Ability to exploit information. These systems will provide medical staff with a powerful research tool which, by combining their clinical observations with supervised and unsupervised machine learning, can be used to develop and test CDSS and other AI functions^4,38^. However, the loss of information from the current CIS prevents the development of these functions. Moreover, it should be noted that, after the COVID-19 global pandemic, the development and use of AI has exponentially increased^39,40^ thanks to its freer availability and ability to integrate large amounts of information about an unknown disease and present it in a simple way to clinicians.

### Our previous experience and justification of this study

In a previous study in ventilator-dependent patients hospitalised at home, we observed the usefulness of the HDDCIS for telemedical real-time patient assessment. Furthermore, the statistical indicators it provides are of great clinical use because they allow the quick and complete analysis of all the information during the morning telemedical rounds^6,35^. However, we were previously unable to validate its use given that specialised healthcare personnel were not available to perform monitoring in domestic settings. In our experience, the application of this technology in the fields of anaesthesiology and intensive care is proving to be extremely useful, especially in the most serious clinical cases, because the resulting monitoring of these patients is more complete and produces fewer artifacts caused by movements, disconnections, or sensor misplacements.

A possible limitation of this study was related to the gold standard used for comparison, given that inaccuracy in the manual recording of vital signs in hospitalised patients has been previously reported^41,42^. However, unlike the random automatic vital sign data-point collections carried out by the current CIS or collected during patient visits by nursing staff in conventional hospitalisation wards^41^, the anaesthesiologist in the operating room and nursing staff in ICUs continuously follow-up and assess patients. Thus, this information is of immense value as it is the most representative of the period in question. Therefore, these registries have formed the basis used to assess the evolution of patients over the last 40 years, thereby facilitating clinical communication and working well in countless clinical studies^9^.

### Discussion of our results

In this current study the perfect correlation we found between the central tendency indicators of both heart rate measurements supports the use of this technology for the purposes set out in this study (Fig. 1). Thus, the values we obtained for manual nursing heart rate measurements (nHR) and the automatic calculations of central tendency (pHR and eHR) were identical and showed very strong correlations and concordances (Tables 2, 3, 4). In addition, the identical correlation of these parameters with the evolution of the acid–base balance—the main pathophysiological alteration present in these patients—indicates that all the clinical value of the nursing records was also captured by the automatic calculations of central tendency. These results indicate that these calculations could replace manual records, thus reducing the bureaucratic burden this task places on staff. Moreover, they allow reliable monitoring measurements to be obtained in settings where staff are not available, as we have already seen in our previous work in home-based settings^6,35^.

In a similar vein, the hourly central tendency calculations for iRR and SpO_2_ were analogous to those for HR, with good (but in this case, not exceptional) correlations with their nursing references. This meant that when the more demanding CCC test (comprising two metrics, one for precision and the other of accuracy) was done, poor values (< 0.9) were obtained and, for reasons intrinsic to the test, these could not exceed their own measure of precision (the Pearson *r* coefficient). These results can be explained in a different way for each parameter. For the iRR, it was related to the movement artifacts that affect this measurement, as discussed below. For SpO_2_, it was related to the low variability of this parameter (especially in these patients without oxygenation problems), which determined the low Pearson correlation coefficient values, regardless of the accuracy and overall concordance of these measurements^43^. Thus, the almost perfect values of its accuracy component (bias correction factor) indicate adequate concordance with the nursing reference values (Table 2).

The clinical evolution by group was as expected, with statistically significant data indicating patient progression towards clinical normality (Tables 4 and 5). Moreover, it was interesting to observe how the decrease in respiratory rate also conditioned a statistically significant decrease in SpO_2_, although this did not translate into clinical significance (Table 4). The logic of this observation is rooted in patient physiology; however, the accuracy of this evolution, reflected both by the nursing records and the hourly statistical calculations, was of far more relevance. Hence, taken together, these current findings also support the use of HDDCIS calculations.

The evolution of the RR was also interesting (Table 5). Movements in these patients, which were initially infrequent while they rested but later increased as they started to recover, likely caused artefacts to appear in the iRR measurements. This could explain the fact that the central tendency calculations initially showed strong Pearson correlations with the nursing reference data, which then became moderate correlations in the improvement group. Thus, these inaccuracies were attributable to the measurement method, not to the data processing. Moreover, this processing ensured that the mode and median still maintained a certain level of clinical utility. Nonetheless, given the importance of this variable, as with HR, we can simultaneously monitor RR using capnography and spirometry as well, thereby allowing us to isolate any discrepancies of this type. Thus, for example, increases only in RR measured by impedance are usually related to the patient's movements.

Finally, we can say that at their highest degree of accuracy, the manual records should match the automatic calculations of central tendency. Therefore, in the absence of major measurement artifacts, we consider that these automatic central tendency calculations should be used as the gold standard for assessing patient evolution.

Like the central tendency parameters, the clinical evolutionary assessment of the minimum (1st percentile) and maximum (99th percentile) hourly calculations was simple for the care staff and was based on the patient age and their clinical situation. Interestingly, despite the selection of these percentiles and the haemodynamic and respiratory stability of these patients, there were significant clinical differences in the HR and RR minimum and maximum hourly calculations with their corresponding nursing references (Fig. 2 and Tables 2, 3, 4, 5). Of note, similar variations were also described for the HR^8^. These findings indicate that automatic random sampling to assess the evolution of these important parameters can differ significantly according to when the sampling is conducted. This could explain, in part, the so-called ‘smoothing effect’. In other words, the trend toward normal or average physiology in the nursing and anaesthesia records^41,42^. Hence, in the operating room and ICU, this smoothing phenomenon could indicate just the opposite: the inaccuracy of random samples and their impaired ability to reflect the true clinical situation of patients. This therefore highlights the need to monitor and modify automatic random registrations^4,8,9^. Thus, given all the above, and in line with these authors, we also believe that random vital sign measurements require supervision, thereby making them inefficient.

## Conclusions

The current CIS discard most of the information generated at the bedside and so the benefits that its storage, processing and exploitation could entail are lost. Furthermore, the random automatic data collection they perform must be supervised by healthcare personnel, making them inefficient. However, a new generation of HDDCIS now being used in anaesthesia and critical care medicine could overcome these limitations. These systems avoid data loss and improve data processing and integration to support the development of more personalised therapeutic regimens. Although more research is still needed to validate this potential for individualising therapeutics, our findings indicate that automatic hourly vital signs calculations of central tendency could replace manual anaesthesia and critical care records, thereby freeing up highly qualified staff for other more demanding tasks.

### Supplementary Information

Supplementary Information.

## Data Availability

The datasets used and/or analysed during the current study are available from the corresponding author upon reasonable request.
