# Supplementary material for: Exploring the clinical relevance of vital signs statistical calculations from a new-generation clinical information system
Source: Sci Rep. 2023 Sep 12;13:15068. doi: 10.1038/s41598-023-40769-3 (PMC10497571; doi:10.1038/s41598-023-40769-3)
Supplement: Supplementary file 1 — Supplementary Information. [file 41598_2023_40769_MOESM1_ESM.pdf]

SUPPLEMENTAL ON LINE CONTENT

EXPLORING THE CLINICAL RELEVANCE OF VITAL SIGNS STATISTICAL CALCULATIONS FROM A NEW-GENERATION CLINICAL INFORMATION SYSTEM

Author's

Juan Ignacio Muñoz-Bonet PhD[*#]; Vicente Posadas-Blázquez MD[*], Laura González-Galindo MD[#]; Julia Sánchez-Zahonero PhD[*], José Luis Vázquez-Martínez PhD[&], Andrés Castillo PhD[§], and Juan Brines PhD[#].

CLINICAL EXAMPLES IN CRITICALLY ILL PATIENTS	Page 2
A. REAL TIME CLINICAL MONITORING WITH THE HDDCIS	
Figure S1	Page 3
Figure S2	Page 4
B. DETECTION AND REPRODUCTION OF CLINICAL EPISODES USING HOURLY STATISTICAL INDICATORS	
Figure S3	Page 5

CLINICAL EXAMPLES IN CRITICALLY ILL PATIENTS

A. REAL TIME CLINICAL MONITORING WITH THIS HDDCIS

The HDDCIS integrated real-time information from the monitoring and treatment equipment in use into one panel which showed the evolution of these data over the 5, 10, 20, 30, or 60 minutes prior. The attending doctor usually has to perform this type of integration in their mind while standing at the patient's bedside by observing the data that fleetingly appears on each piece of equipment connected them. Thus, there is no record of the doctor's estimations, nor can the data be reproduced later because this information is scattered among several devices and easily gets lost.

Figure S1 shows the clinical evolution (over the 10 minutes prior) of an infant on invasive mechanical ventilation due to severe bronchiolitis (pressure control ventilation). Note how the drop in their tidal volume (arrow on the yellow line) caused a drop in oxygen saturation (to 78%) 2 minutes later (arrow on the pink line) and an increase in end tidal CO_2 (up to 64 mmHg) (arrow on the orange line). These parameters were normalised by increasing the ventilator pressure by 3 cmH_2O (arrows on the dark blue line of the lower graph). Because the strict alarm programming of multiple independent parameters leads to an unacceptably high number of false positive alarms which affect patients, families, and caregivers (10), these episodes typically go undetected until they cause a significant vital sign disturbance (as in this case). A quick glance at these graphs allows early diagnosis of these events (in this case, 2 minutes earlier), thereby helping to avoid patient deterioration.

Figure S2. shows the clinical evolution (over the 60 minutes prior) of an adolescent weighing 60 kg on invasive mechanical ventilation because of severe acute respiratory distress syndrome (volume control ventilation). In this episode we can see the stable situation of the patient prior to their mobilisation for the placement of an anti-decubitus mattress (in the area highlighted on the graphs) as well as the clinical consequences of this manipulation.

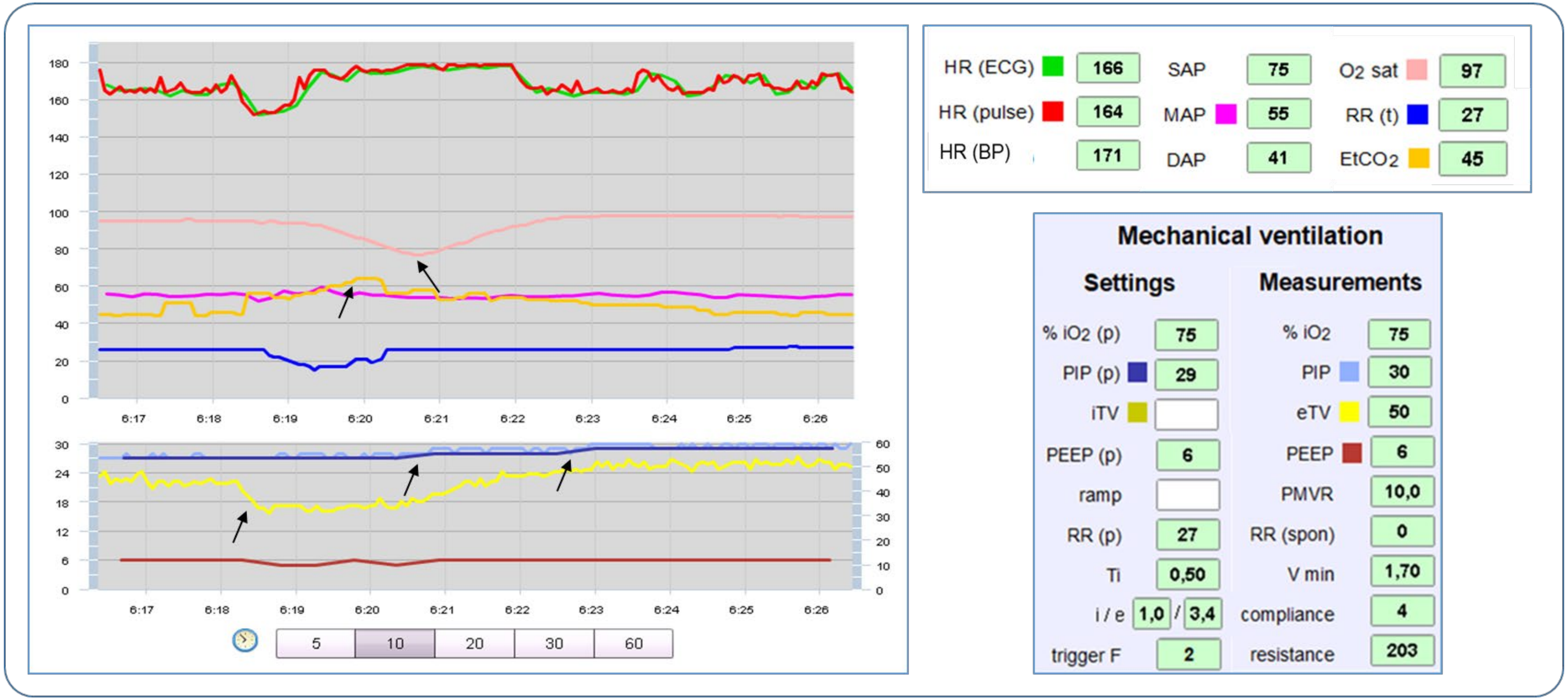

Figure S1. The real-time evolution of a ventilated infant with severe bronchiolitis. (1) Left: superimposed graphs (on the same time axis) of vital sign (top) and mechanical ventilation (bottom) data; a colour-code legend for each parameter represented in the graphs is shown in the tables. The vital signs graph has a common automatic adjustment scale for all the parameters (0–180) and, in this case, encompasses the 10 minutes prior for the following parameters: *heart rate* measured by electrocardiogram (HR (ECG), green line), heart rate measured by pulse oximetry (HR (pulse), red line), *oxygen saturation* (O_2 sat, pink line), *mean arterial pressure* (MAP, magenta line), *end-tidal carbon dioxide concentration* ($EtCO_2$, orange line), and *respiratory rate* (RR(t), dark blue line). The mechanical ventilation graph has a double automatic adjustment scale; in this case, the pressure scale shown on the left (0–30 in cmH_2O) and volume scale on the right (0–60 mL) include the following parameters: *peak inspiratory pressure programmed* on the ventilator (PIP(p), dark blue line), *peak inspiratory pressure* measured by the ventilator (PIP, light blue line), ventilator-measured *expiratory tidal volume* (eVT, yellow line), and *positive end-tidal pressure* (PEEP, maroon line). (2) Right: tables with the real-time parameter values corresponding to the values on the right-hand limit of the graphs.

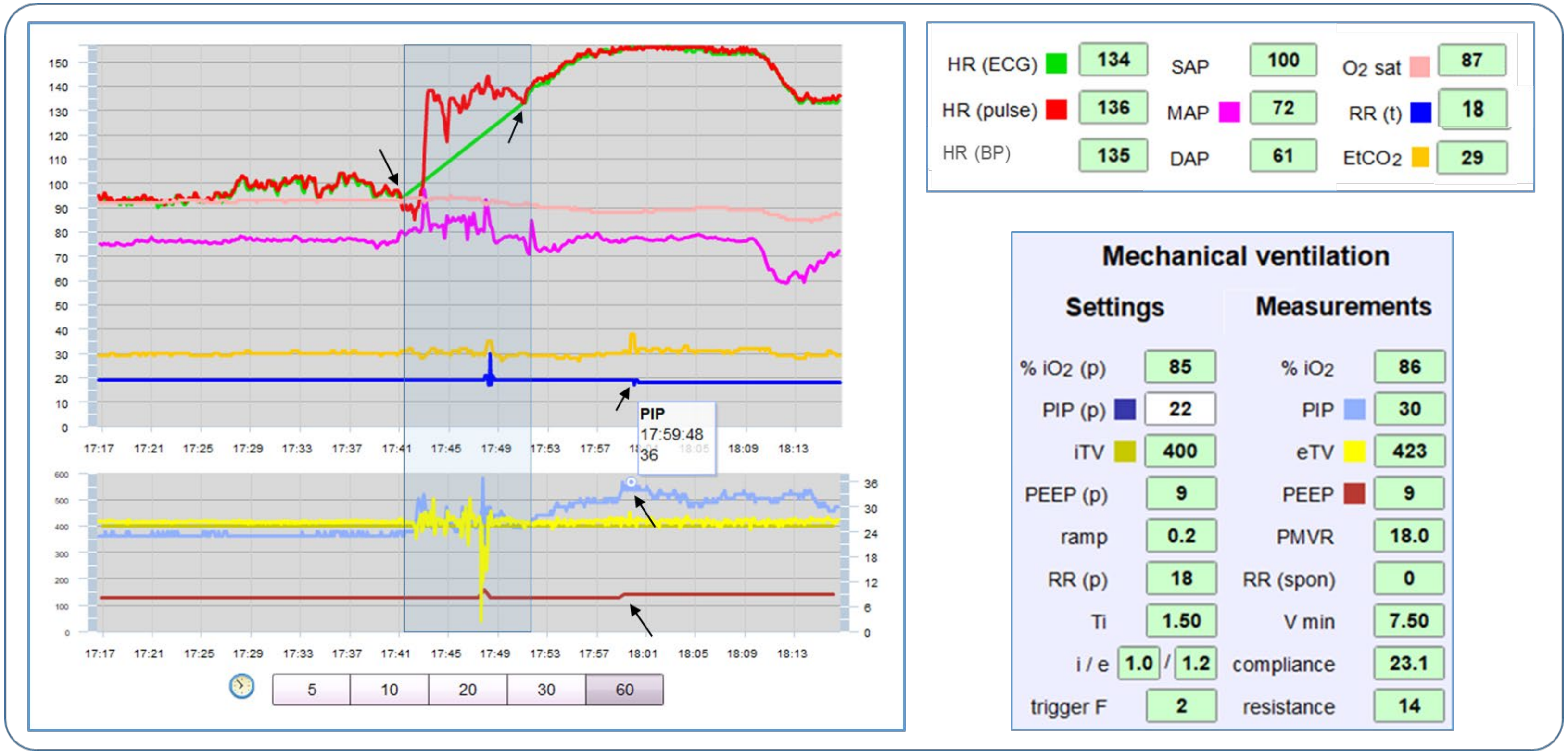

Figure S2. The real-time evolution of a ventilated patient with severe acute respiratory distress syndrome. These graphics can be interpreted following the same logic explained in Figure 1 for the vital signs and mechanical ventilation graphs. A colour-code legend for each parameter represented in the graphs is shown in the tables. Note how: (1) the patient was on volume-controlled mechanical ventilation (settings column: inspiratory tidal volume [iTV] LED activated), without any respiratory effort recorded during the entire period (the total respiratory rate [RR(t)] was equal to the programmed respiratory rate [RR(p)] and the spontaneous respiratory rate [RR (spon)] was 0). (2) Prior to mobilisation, the patient was stable on deep sedoanalgesia and muscle relaxation, requiring a peak inspiratory pressure (PIP) of 23–24 cmH_2O to deliver the 400 mL of programmed gas (compliance was 30–31 mL/cmH_2O). (3) Their manipulation in order to place an anti-decubitus mattress (shown in the highlighted area on the graphs) conditioned a significant and sustained increase in the heart rate and a progressive desaturation (up to 85%) with an increase in the PIP by up to 36 cmH_2O (arrow over light blue line). This was the result of derecruitment following the mobilisation (a fall in compliance by 16 mL/cmH_2O), despite not being disconnected from the mechanical ventilator as indicated by the evolution of the PIP, PEEP and EtCO_2. (4) Monitoring of the electrical heart rate [HR (ECG)] was lost while the patient was being mobilised because of electrode disconnection (arrows on the upper graph on the red and green lines). (5) After mobilisation, the medical team increased PEEP from 8 to 9 cmH_2O and decreased respiratory rate from 19 to 18 rpm (arrows on bottom graph maroon line and on the upper graph on the blue line, respectively). Note: the mechanical ventilation graph has a double automatic adjustment scale; in this case, the volume scale is shown on the left (0–600 mL) and the pressure scale is shown on the right (0–36 in cmH_2O).

B. DETECTION AND REPRODUCTION OF CLINICAL EPISODES USING HOURLY STATISTICAL INDICATORS

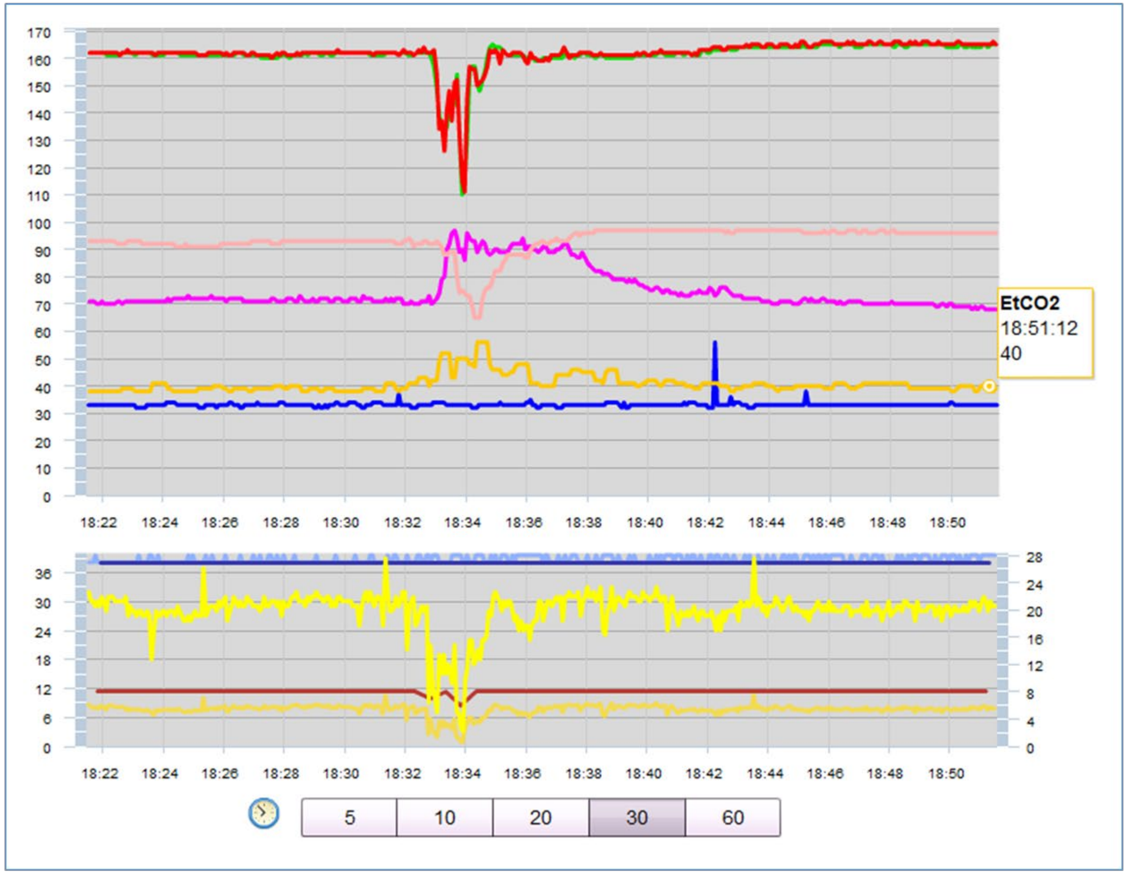

	^15	16	17	18	19	20	^21
HR (pulse)							
Maximum	164	162	162	167	174	170	170
Mode	157	157	159	162	166	168	168
Minimum	156	155	157	145	164	164	166
Std. deviation	2	2	1	4	3	1	1
HR (ECG)							
Maximum	164	161	161	166	174	170	170
Mode	157	157	159	161	166	166	167
Minimum	156	154	157	145	164	164	166
Std. deviation	2	1	1	4	3	1	1
O2 sat							
Maximum	98	98	97	97	97	97	98
Mode	96	95	94	93	96	95	97
Minimum	92	93	92	88	89	94	93
Std. deviation	1	1	1	4	1	1	1
RR (t)							
Maximum	34	34	34	34	34	34	33
Mode	33	33	33	33	33	33	33
Minimum	32	32	32	32	32	32	33
Std. deviation	0	0	0	1	1	0	0
PIP							
Maximum	28	28	28	29	29	29	29
Mode	27	27	27	27	29	29	29
Minimum	26	27	27	27	28	28	28
Std. deviation	1	0	0	1	0	0	0
EtCO2							
Maximum	62	45	45	56	51	42	35
Mode	41	38	38	38	38	35	32
Minimum	36	36	36	37	35	30	30
Std. deviation	4	2	2	3	2	3	1

Figure S3. Desaturation episode in an infant with severe bronchiolitis after manipulation (a diaper change). Real-time evolution Graph (left) and Table with hourly statistical calculations (right). Note how (1) patient handling conditioned a significant drop in the *expiratory tidal volume* (lower yellow line), which caused a drop in *oxygen saturation* (pink line) and an increase in *end-tidal* CO_2 (orange line) with the typical 'box' morphology of increased resistance in the patient's airway (6). (2) The haemodynamic repercussions of the episode were expressed over the baseline as a brief bradycardia (superimposed lines for the *ECG-HR* in green and *pulse oximetry-HR* in red) and a rise in *mean blood pressure* (magenta line) for more than 5 minutes. (3) The episode can be easily identified by estimating the hourly standard deviation (Std. deviation) of the *oxygen saturation* (row highlighted in blue in the table. Normal value ≤ 1). (4) The basal *heart rate* was fixed (graph and low basal Std. deviation-HR values), which was an expression of the serious condition of the patient. The fixed *respiratory rate* (RR(t)) in the graph (upper blue line) and in the table indicated that the patient was under controlled mechanical ventilation without *respiratory rate* modification during this period.
